# Effect of vitamin D3 supplementation on TNF-α serum level and disease activity index in Iranian IBD patients

**Published:** 2015

**Authors:** Tahereh Dadaei, Mohammad Hosein Safapoor, Hamid Asadzadeh Aghdaei, Hedie Balaii, Mohammad Amin Pourhoseingholi, Nosratollah Naderi, Homayoon Zojaji, Pedram Azimzadeh, Parvaneh Mohammadi, Mohammad Reza Zali

**Affiliations:** *Gastroenterology and Liver Disease Research Center, Shahid Beheshti University of Medical Science, Tehran, Iran*

**Keywords:** Inflammatory bowel disease, TNF-α, Vitamin D

## Abstract

**Aim**: The aim of the study was to assess the effectiveness of vitamin D_3 _[1, 25(OH)_2_D_3_] treatment in IBD with regard to tumor necrosis factor-alpha (TNF-α) serum level and clinical disease activity index (CDAI).

**Background**: Vitamin D has immune-regulatory functions in experimental inflammatory bowel disease (IBD) and vitamin D deficiency is common in IBD patients.

**Patients and methods**: This was a randomized clinical trial on 108 IBD patients with serum 25-OHD levels less than 30ng/ml, which divided into vitamin D and control groups. Vitamin D group received 50000 IU vitamin D_3_ for 12 weeks. Before and after the study, TNF-α and 25-OHD serum levels were measured by ELISA method. Data were analyzed using paired t-test, chi-square test and Spearman correlation coefficient. P-values ​​less than 0.05 were considered statistically significant.

**Results**: Before the intervention no significant difference was found between baseline characteristics and TNF-α serum level of two groups. After intervention TNF-α serum level reduced but this reduction was not statistically significant (p= 0.07, 95% CI: -0.45 to 8.14). The mean serum 25-OHD level of vitamin D increased from 15.54 to 67.89, which was statistically significant (p= 0.00, 95% CI: -61.40 to -43.30). TNF-α level was also associated significantly with CDAI before (Spearman’s rho: 0.3, p<0.0001) and after (Spearman’s rho: 0.27, P=0.01) intervention.

**Conclusion**: Oral supplementation vitamin D_3_ significantly increased serum vitamin D levels and insignificantly reduced serum TNF-α level. More studies with larger samples would be beneficial to assess vitamin D_3_ supplementation efficient effect in IBD.

## Introduction

The inflammatory bowel diseases (IBDs), Crohn's disease and ulcerative colitis, are chronic inflammatory disorders of the gastrointestinal tract. These diseases characterized by aberrant mucosal immune responses to intraluminal antigens in genetically predisposed individuals (1). TNF-α and several other pro-inflammatory cytokines, play a main role in the induction and perpetuation of intestinal inflammation in IBDs so that it was shown that treatments against these cytokines particularly anti-TNF-α antibody are effective in IBD patients (2).

In addition of genetics and immune factors, environmental factors affect the development of IBD. Twin studies have shown not all people who are genetically predisposed to develop IBD, actually develop IBD and epidemiologic studies have examined numerous environmental factors that alter the expression of the genes and can control IBD development in genetically susceptible subjects (3-5).

Epidemiological studies in IBD demonstrate a more prevalence in northern climates such as Northern Europe and North America (6). Multiple sclerosis (MS) and type-1 diabetes, two major autoimmune diseases, are similarly most prevalent in countries far from equator, in Scotland and Finland, respectively. Vitamin D available from sunlight exposure is significantly less in these climates, which has been suggested to be a contributing factor to IBD (7, 8). 1,25-Dihydroxyvitamin D_3 _[1,25(OH)_2_D_3_], the bioactive form of vitamin D, has well-known effects on calcium metabolism, bone formation and mineralization. In 1983, the vitamin D receptor (VDR) was discovered in human leukocytes and after that, its identification in skin, placenta, pancreas, breast and other tissues that are not involved in maintaining mineral homeostasis and bone health, has provided new insights into the function of this vitamin as an important immune system regulator (9,10,11). In addition, genome-wide screening techniques suggest that VDR polymorphisms are associated with increased susceptibility to both Crohn's disease and ulcerative colitis in human subjects (12,13).

Vitamin D and VDR deficiency have been shown to exacerbate experimental IBD and treatment of mice with 1,25(OH)_2_D_3 _inhibits the development of disease (14). Vitamin D and VDR are needed for normal function of two type of regulatory T cells include iNKT cells and CD4/CD8αα intraepithelial lymphocytes. The experimental evidence show that in the absence of vitamin D and VDR, IBD occurs by changes in vitamin D status and VDR signaling and increase the amount of some proinflammatory cytokine production including tumor necrosis factor-α (TNF-α) and interferon-γ (15). Furthermore, animal models suggest that vitamin D is positively involved in maintaining or restoring immune homeostasis, which might be useful in the treatment of IBD. However, it should be noticed that human effects might differ from animal effects due to the interaction between different vitamin D sensitive cells (16). Vitamin D supplementation trials will further elucidate the immune regulating effects of vitamin D on the immune system of humans.

The aim of this study was to assess the effectiveness of vitamin D treatment in IBD with regard to reduction of TNF-α level and disease activity index. 

## Patients and Methods

**Study design and participants**

The study was a randomized controlled trial, carried out at Taleghani Hospital, Tehran. The study was approved by Research Center for Gastroenterology and Liver Disease Ethics Committee and was registered in the Iranian Registry of Clinical Trial (www.irct.ir) with registration number ID: IRCT2013122915686N1.

Patients with confirmed inflammatory bowel disease by a gastroenterologist according to established clinical guidelines and criteria based on endoscopic, radiological and histopathological examination were recruited into the study. Exclusion criteria included patients with a, serum 25OHD level more than 30ng/ml, confirmed celiac disease, kidney disorders requiring dialysis or polycystic kidney disease also pregnant patients. Written informed consent was obtained from all patients. 

**Procedures**

The patients were randomly divided into treatment and control group with random block design method. The information was obtained using a questionnaire consisting of demographics, occupation, medical history, smoking, past and current medication. Medication use was classified as ever having used of 5-aminosalicylic acid compounds, immune modulators (ie, azathioprine, 6-mercaptopurine, and methotrexate) and anti-tumor necrosis factor (TNF) biologic agents (ie, infliximab, adalimumab). Disease activity was measured using the ulcerative colitis disease activity index (UCDI) in UC patients and the Crohn's disease activity index (CDAI) in CD patients (17, 18). Moreover, the patients in treatment group received oral vitamin D (cholecalciferol; 50000 units/week) for 12 weeks, while the control group received no experimental treatment. Serum samples were stored at -70˚C until each participant completed the study. Serum 25-hydroxy vitamin D levels were measured using an enzyme immunoassay (25-hydroxy vitamin DEIA kit; Immunodiagnostics Systems, Boldon, UK) with a measurement sensitivity of 5 nmol/l, and an intra- and inter-assay coefficient of variation of 5.9% and 5.1%, respectively. Also, serum TNF –α level was measured with ELISA method by Human TNF alpha ELISA Ready-SET-Go kit produced by eBioscience company (The United States).

**Statistical analysis**

Continuous variables were expressed using means and standard deviations. The chi-square test and the t-test were used to perform between-groups comparison for categorical and continuous variables, respectively. Moreover, for comparisons within groups, before and after the treatment, paired t-test was performed. The association between quantitative variables was estimated by the Spearman coefficient of correlations. P-values of less than 0.05 were assumed to be significant (p<0.05). 

## Results

**Figure 1:**
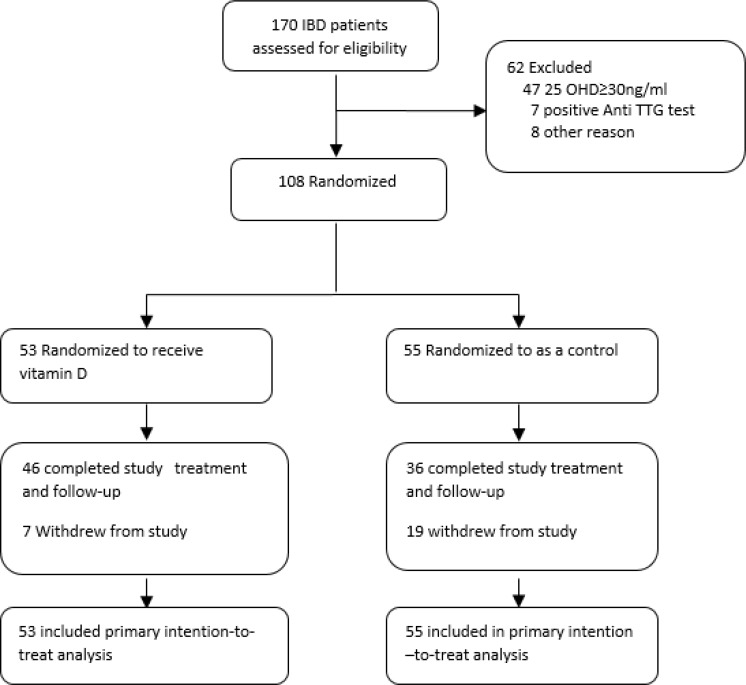
Participant Flow Chart

Of 170 potential participants for inclusion criteria, 108 were eligible and were randomized to vitamin D or control groups. 82 participants (76%) completed the study treatment and follow-up, 19 (17.5%) in control group and 7 (6.5%) in vitamin D group withdrew from the study. 108 participants were included in the intention-to-treat analysis. The flow of participants through each stage of the study is shown in figure 1. 

Both groups were similar in age, sex and other baseline characteristics before intervention (table 1). The mean age was 37.36±14.69 years and 38.75±15.73 years in the vitamin D and control groups, respectively and the mean duration of disease was 60.07±58.41 months and 65.74±58.26 months in the vitamin D and control groups, respectively. 

Baseline TNF-α levels did not differ between the vitamin D group and control group [mean TNF-α 33.3 pg/ml (S.D. 29.3 pg/ml) vs. 28.0 pg/ml (S.D. 20.1) p=0.27]. After intervention serum TNF-α levels decreased from mean 33.3 (S.D. 29.3 pg/ml) to mean 26.64 (S.D. 17.56 pg/ml) but this difference was not statistically significant (p=0.07, 95% CI: -0.45 to 8.14).

At the beginning the mean 25OHD levels in control group was more than vitamin D group significantly, while at the end this mean in vitamin D group was significantly more than control group (p=<0.0001, 95% CI: -61.40 to -43.30). Therefore, in vitamin D group the mean 25OHD levels from 15.54 (S.D. 7.7) increased to 67.89 (S.D. 33.7) but on other side it increased from 21.70 (S.D. 7.8) to 23.90 (S.D. 8.3) in control group.

TNF-α level was also associated significantly with CDAI (clinical disease activity index) before (Spearman’s rho: 0.3, p<0.0001) and after intervention. (Spearman’s rho: 0.27, p=0.01).

## Discussion

Noticeable epidemiologic data have shown a correlation of autoimmune disease with sun exposure and decreased vitamin D status. Living at higher latitudes increases the risk of type-1 diabetes, multiple sclerosis, cancers and IBD (19). Immigration from high-risk areas before puberty reduces the risk of multiple sclerosis by approximately 50% (20). However, vitamin D deficiency is common in patients with IBD even when the disease is in remission (21). In our study, vitamin D deficiency or insufficiency was found in 119 out of 170 (70%) IBD patients. Some evidence supports the vitamin D supplementation in the treatment of some immune-related disease such as IBD, is increasing. The origin for vitamin D use as an adjuvant therapy in IBD is based on experimental animals and in vitro studies. These studies express that vitamin D has potent immune-regulatory functions and is assumed to be critical to maintain a balanced immune response. As a result, it reduces the pro-inflammatory condition observed in IBD (22, 23). 

**Table 1:** Baseline characteristics of vitamin D and control group

	Vitamin D	Control	P-Value
Disease type, n(%)			0.28
Ulcerative colitis	43(81.1)	49(89.1)	
Crohn's disease	10(18.9)	6(10.9)	
Age (years), mean(S.D)	37.3 (14.7)	38.7 (15.73)	0.63
Sex, n(%)			0.56
Female	27(50.9)	32(58.2)	
Man	26(49.1)	23(41.8)	
Disease duration(month), mean(S.D)	60.1(58.4)	65.7 (58.3)	0.61
Illeal Disease, n(%)			0.23
Yes	2	0	
No	51	55	
Smoking Status, n(%)			0.37
Current	46(86.8)	42(76.4)	
Ex-Smoker	3(5.7)	6(10.9)	
Never	4(7.5)	7(12.7)	
Azathioprine users, n(%)			1.00
Yes	22(41.5)	23(41.8)	
No	31(58.5)	32(58.2)	
Baseline TNF-α level, mean(S.D)	33.3 (29.3)	28.0 (20.1)	0.27
Baseline25OHD, mean (S.D)	15.5 (7.7)	21.7 (7.8)	0.04
Baseline CDAI*			
Active	22(41.5)	21(38.2)	0.84
Remission	31(58.5)	34(61.8)	

* CDAI: Clinical disease activity index

In the present study, we investigated the effects of vitamin D_3_ treatment on reduction TNF-α (one of the most important pro-inflammatory cytokine in IBD) levels and clinical activity disease index (CADI) in IBD patients. Oral intake of 50000 IU vitamin D3 once weekly for 12 weeks, significantly increased serum 25OHD levels from 15.54 to 67.89 (p= 0.00). Also, it reduced the TNF-α level from 33.3% to 26.64% but this reduction was not statistically significant (P=0.07). 

Few clinical trials have been undertaken regarding the effect of vitamin D administration in IBD course and to our knowledge; this is one of the first clinical trials to estimate the effect of vitamin D3 treatment on IBD disease course with regarding to reduction of TNF-α level and CDAI. 

In a pilot study on a limited number (n=18) Crohn's patients by Yang et al. vitamin D oral supplementation significantly increased serum 25-OHD levels and reduced the unadjusted mean CDAI (Crohn's disease activity index) scores. In this study markers of inflammation associated with disease such as CRP, ESR, TNF-α, IL-17, IL-10 and vascular endothelial growth factor was assessed before and after vitamin D_3_ treatment but all of them didn’t found to be affected by the vitamin D_3_ supplementation (24). A large part of these results were similar to our results.

A meta-analysis study by Nicholson et al. in 2012 has reported that only four clinical trials (two of them examined human participants and the other two included experimental animals) have evaluated the effect vitamin D supplementation in inflammatory bowel disease (25). All of them demonstrate some benefits to use the vitamin D supplements but only two of these studies (animal’s studies) had statistically significant results (26,27). Of course the results from animal studies are limited in their application to the human species. 

The clinical trial by Jorgensen et al. reported a non-significant reduction of relapse rate for Crohn's disease compared to placebo (29% vs. 13%, P=0.06) (28). Also, another human clinical trial by Miheller et al. demonstrated the effects of one form of vitamin D has more effects in maintaining disease remission compared to the other form at the end of 6 weeks of the trial but not at the end of 12 months (29). 

However, two retrospective cohort studies in inflammatory bowel disease found statistically significant results. In the first study, had examined vitamin D status influences durability of anti-tumor necrosis factor (TNF)-α therapy in patients with IBD. The findings showed patients with insufficient vitamin D because of the lack of response to treatment stopped anti-TNF-α therapy earlier than sufficient subject (30).

Moreover, in another retrospective cohort study Ultisky et al. showed that vitamin D deficiency was associated with higher disease activity (31). However, there are some limitations in retrospective cohort studies. As the information was not specifically gathered for research purpose thus these studies may remind bias. 

Current study also has some limitation such as the short-term period of treatment. Extending the treatment duration may help more comprehensive evaluation of the effects of vitamin D. The other limitation of our study is the lack of use of a placebo, given that the number of patients who were excluded from the control group more than the number of missing vitamin D group patients. This study as the first study about the association of vitamin D deficiency and inflammatory bowel disease in Iran has some strength points including relatively large sample size and the recruitment inclusion criterion of vitamin D deficiency or insufficiency. 

The present study showed oral supplementation vitamin D_3_ significantly increased serum 25OHD levels and insignificantly reduced serum TNF-α level. However, more studies with larger samples would be beneficial to assess vitamin D_3_ supplementation efficient effect in IBD.
